# Incidental Ureteroinguinal Hernia in a Patient With a Single Kidney

**DOI:** 10.5334/jbsr.2802

**Published:** 2022-05-06

**Authors:** Ana Costa, Joana Granadas, Marta Baptista

**Affiliations:** 1 Hospital Prof. Doutor Fernando Fonseca, E.P.E., PT

**Keywords:** hernia, ureter, inguinal

## Abstract

**Teaching point:** An ureteroinguinal hernia is a very rare diagnosis with typical radiological findings that must be reported, particularly in surgical candidates.

## Case History

**Figure 1:**
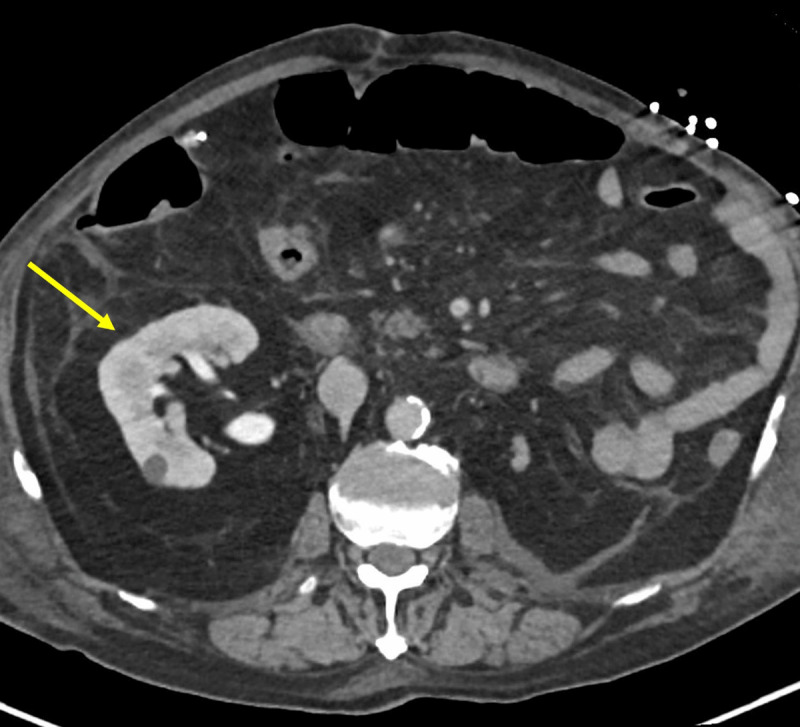


**Figure 2:**
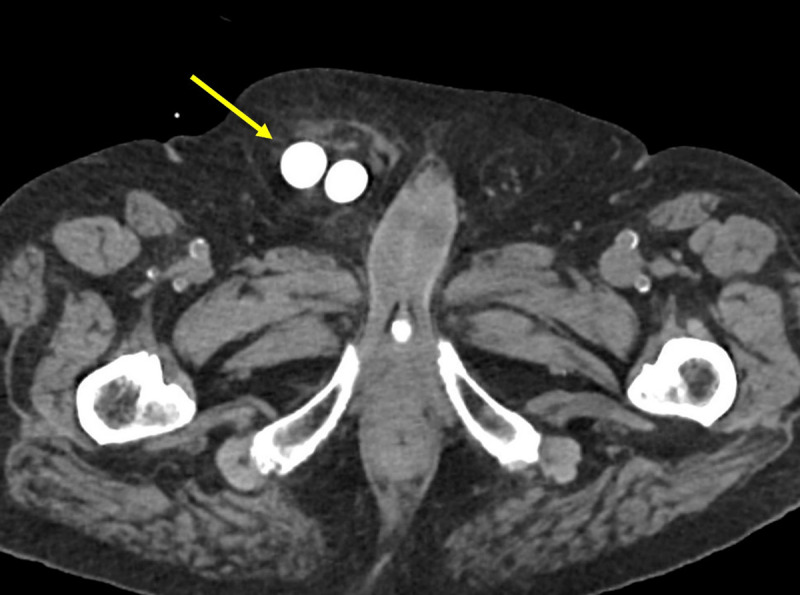


**Figure 3:**
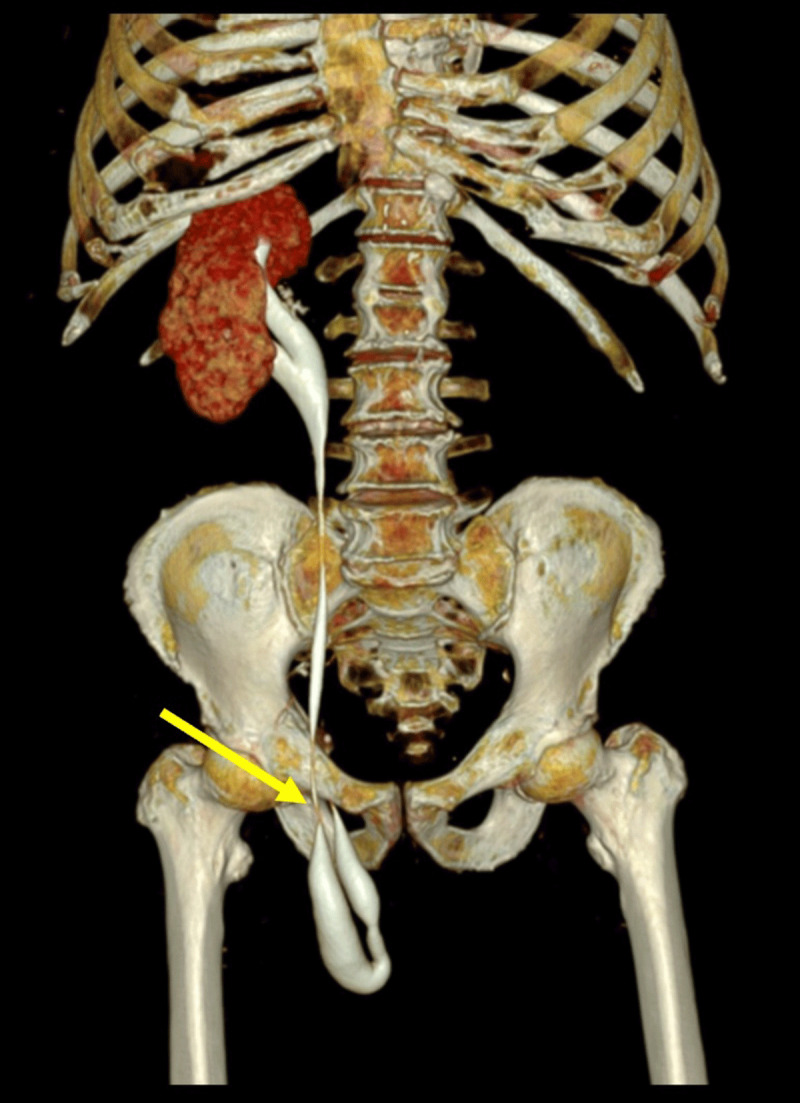


An 86-year-old man with hypertension, type II diabetes, and no surgical history was admitted for acute upper gastrointestinal bleeding due to a bulbar ulcer. For clinical worsening after the upper endoscopy, an abdominopelvic computed tomography (tube voltage 140 kV; tube current 428 mAs) with intravenous contrast (80 ml 300 mg iodine/mL, 4 mL/s) on arterial, venous, and urographic phases (25, 70, and 180s after injection, respectively) was performed. Images showed signs of gastrointestinal perforation. Incidentally, a right single kidney was detected (***Figure 1***, arrow on axial image) with an incomplete duplex collecting system, with a bifid pelvis and a single ureter. The ureterovesical junction was normally positioned. Hydroureteronephrosis was present, and the ureter descended into the right inguinoscrotal region (***Figure 2***, arrow on axial image), with stenosis at the hernia defect (***Figure 3***, arrow on 3D coronal reformation). Renal function tests showed high creatinine (1,50mg/dL) and low glomerular filtration rate (47 ml/min/1,73 m^2^). The patient underwent surgery for ulcerorrhaphy, during which a right indirect inguinal hernia with peritoneal fat was found. The ureter was in a retroperitoneal location, accompanying the hernia. The final diagnosis was a paraperitoneal ureteroinguinal hernia. This was not surgically approached over hemodynamic instability. The patient improved, renal function tests normalized, and the hydroureteronephrosis decreased at follow-up imaging.

## Comment

An inguinal hernia is a common diagnosis, frequently leading to surgery. However, inguinal herniation of the ureter is an extremely rare finding in patients with native kidneys when there is no associated ectopic or transplanted kidney. Risk factors include male sex, age above 50 years old, collagen synthesis deficiencies, obesity, and previous kidney transplantation. This is a late complication after transplantation and must be sought when there is hydronephrosis to prevent graft loss. Previous hernia repair is not an established risk factor. Patients may have urinary symptoms, obstructive uropathy, or be asymptomatic. Nowadays, ultrasound, computed tomography (CT), and magnetic resonance imaging (MRI) can perform the diagnosis. The classic “curlicue” or “loop-the-loop” sign can be seen on reconstructed CT and MRI images, representing a redundant loop of the ureter and its abrupt change in calibre as it passes through the hernia defect. Urographic phase images are crucial in doubtful cases. There are two types of ureteroinguinal hernia. The paraperitoneal type (80% of cases) is acquired and is a sliding hernia, as the herniation sac drags the ureter due to adhesions to the parietal peritoneum, frequently along with viscera. Hydroureteronephrosis develops as there is a battle for space at the hernia defect. These are generally indirect hernias, large, reducible, and asymptomatic. The extraperitoneal type (20% of cases) is congenital and lacks a peritoneal sac. The ureter herniates along with retroperitoneal fat because insufficient ureteral differentiation from the Wolffian duct leads to traction of the ureter through the persistence of genitoinguinal ligaments. These hernias are usually minor defects, nonreducible, and symptomatic. They are commonly associated with renal or ureteral malformations. Ureteroinguinal hernias are most frequently found at the time of surgery. The same surgical techniques used in inguinal hernia repair can be employed in ureteroinguinal hernias, but placement of a ureteric stent is recommended to localize better and protect the ureter [1].

## References

[1] Beebe, K, Muhonen, J, Giuseppuci, P, Esper, C. A rare case of an inguinal hernia-containing (extraperitoneal) ureter. Am J Case Rep. 2021; 22: e930911. DOI: 10.12659/AJCR.930911 34489391 PMC8436827

